# Fluoroscopy Guided Cervical Plexus Block for Carotid Endarterectomy - A Case Report

**Published:** 2009-06

**Authors:** Aparna A Nerurkar, Vandana V Laheri, Hemangi S Karnik, Shubha N Mohite

**Affiliations:** 1 Associate Professor, Department Of Anaesthesiology, Lokmanya Tilak Municipa Medical College and, Lokmanya Tilak Municipal General Hospital; 2,3 Professor, Department Of Anaesthesiology, Lokmanya Tilak Municipa Medical College and, Lokmanya Tilak Municipal General Hospital; 4 Professor And Head, Department Of Anaesthesiology, Lokmanya Tilak Municipa Medical College and, Lokmanya Tilak Municipal General Hospital

**Keywords:** Cervical plexus block, Fluoroscopy, Carotid endarterectomy

## Abstract

**Summary:**

Carotid endarterectomy(CEA) is being increasingly performed under regional anaesthesia supplemented with sedation, the world over. Deep or superficial cervical plexus blocks or a combination of both have been found to be equally effective. Various imaging modalities like fluoroscopy, computed tomography (CT), CT-fluoroscopy, ultrasound etc have been used to increase the success rates of the technique and to reduce the rate of complications associated with the block. These are especially useful given the varying landmarks quoted by various authors as also inter-individual differences in anatomy. We present a case report of how fluoroscopy aided us in administering cervical plexus block.

## Introduction

Regional anaesthesia in the form of deep or superficial cervical plexus block or a combination of both is gaining popularity as technique of choice for carotid endarterectomy^1^‐^4^. An important advantage is that it allows continuous monitoring of patient's neurological status thus ensuring timely therapeutic intervention. Being a blind technique, its success is limited by the land-marks one follows and the case to case variation in the cervical anatomy. Success rates are increased when imaging modalities are used to supplement the performance of the block. We present a case where fluoroscopy aided us in the placement of a successful deep cervical plexus block.

## Case Report

Pre-operative assessment: A 35-year-old, 75 kg male, with a 5 week history of right-sided hemiparesis, right-sided facial lower motor neuron palsy with Broca's aphasia was posted for carotid endarterectomy. Pre anaesthetic assessment revealed history of hypertension controlled with amlodipin 5 mg BD since 1 year, occasional alcohol intake and 5 pack years of smoking. Examination confirmed the neurological diagnosis and revealed no other systemic abnormality. Preoperative hematological, biochemical investigations, chest skiagram and electrocardiogram were within normal limits. Plasma homocysteine levels were more than 50 micromole/deciliter as compared to the normal range of 5-12 micromole/deciliter for our laboratory. MRI angiography and carotid Doppler revealed 83% stenosis of left internal carotid artery at the level of its origin with left middle cerebral artery infarct.

Procedure: Deep cervical plexus block with sedation was planned for the procedure. Informed consent was obtained after explaining the risks and benefits to the patient. After confirming nil per oral status, patient was taken on table and routine monitoring with pulse-oximeter and cardioscope was commenced. Baseline vital parameters were recorded and peripheral intravenous access was obtained with an 18 gauge indwelling intravenous cannula. Patient was premedicated with intravenous glycopyrrolate 0.2 mg, midazolam 1 mg and fentanyl 75 mcg. Oxygen was administered at the rate of 6 litres/minute using Hudson's mask. Arterial and central venous lines were secured under local anaesthesia. Patient was catheterized to prevent discomfort due to bladder distension. Special attention was given to maintenance of temperature by using warm intravenous fluids, warming mattress and convective forced air blowing system.

**Fig 1:**
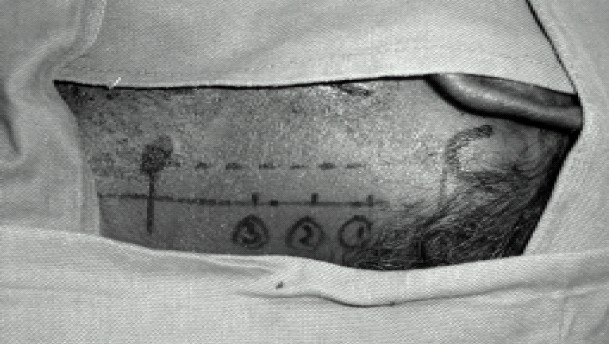
Landmarks for deep cervical plexus block

**Fig 2:**
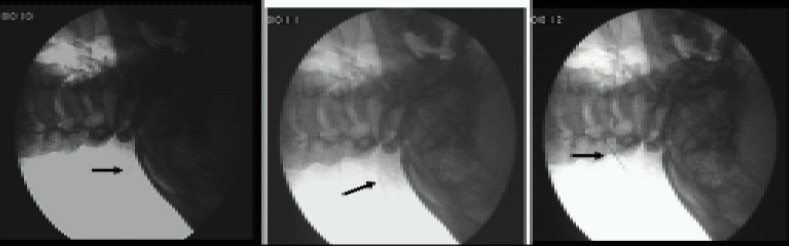
Sequence of needle pointing to C1, redirected to C2 and on transverse process of C2

Patient was positioned supine with head turned to right side. Mastoid process and Chassaigne's tubercle were identified and a line was drawn joining the two points. Another line was drawn parallel and about 1 cm posterior to this line. Points were marked 2 cm, 4cm and 6 cm caudal to the mastoid process on the second line (Fig 1). A 5 cm 22 gauge needle was inserted at the first point, 2cm caudal to the mastoid process with the needle directed perpendicular to the skin and fluoro-scopic imaging done to confirm the position. It was found that needle was directed towards C1 (Fig 2). The needle was withdrawn and redirected caudad and the transverse process of C2 was contacted at a depth of approximately 2cm (Fig 2). After confirming fluoroscopically and clinically 4 cc of a mixture of 1% lidocaine and 0.25 % bupivacaine was injected after withdrawing the needle slightly. Similar technique was used to confirm the transverse processes of C3 and C4 and 4 cc of the same mixture was injected at each point.

Onset of anaesthesia was checked with loss of sensation to pin prick over corresponding dermatomes on the same side of the neck - skin of the anterolateral neck and the ante- auricular and retro-auricular areas. Surgery could be commenced in 15 min. The procedure lasted 3 and ½ hours and patient needed supple-mentation with 2% lidocaine and 0.5% bupivacaine around the carotid sheath during sheath dissection. Additional boluses of midazolam and fentanyl to a total of 3 mg and 300 mcg respectively were given as per patient's requirements. Apart from above mentioned monitoring, hourly arterial blood gases were monitored which were within normal limits. Vitals were stable except for a rise in systolic pressure by 20 mm Hg during clamping which lasted for 45 minutes. Patient was assessed for neurological impairment during this stage and no fresh deficits were noted. Post operative course in intensive care setup was unremarkable and slow neurological improvement was seen in follow up visits.

## Discussion

CEA is being increasingly performed under regional anaesthesia. Cervical plexus blocks in the form of superficial plexus blocks or deep cervical plexus blocks or a combination of both with or without supplemental analgesia and sedation offer good results. The success of the block often depends on accurate placement and optimised delivery of the local anaesthetic. Landmarks for the block placement are therefore of immense importance. However the landmarks quoted by various authors and institutes differ significantly. NYSORA^5^ (New York School Of Regional Anesthesia) marks the injection points at 2, 4 and 6cm caudal to mastoid on the line connecting the mastoid and C6 tubercle, UNC^6^ (University of New Carolina) marks them on a line parallel and 1 cm posterior to this line while other textbooks^7^,^8^ suggest a line 0.5 cm posterior to the same line. The Osler Institute^9^ suggests a line between the mastoid process and the suprasternal notch while the University of Palermo^10^ recommends localisation of C6 and C4 first, followed by drawing a line 1 cm posterior to the mastoid and finding the intersection of this line with the line marking the C4 level.

With a plethora of landmarks and inter individual variability, it would be prudent to use various imaging modalities or nerve locators to give the block safely. The techniques also aid in increasing the success rate of blocks by ensuring correct needle placement and delivery of the drug. These adjuncts gain special importance with a single injection technique^6^,^11^ as compared to multiple injections technique.

Fluoroscopic imaging facilities may be available as part of neurosurgical intervention complexes, as in our institution, and should be used when available. Our case experience definitely demonstrates its utility. Other more accessible alternatives include Ultrasonography guided^12^ and nerve locator^13^ guided blocks. They will improve the success and safety and consequently the acceptability of regional technique.
